# Policy content and stakeholder network analysis for infant and young child feeding in Nepal

**DOI:** 10.1186/s12889-017-4340-6

**Published:** 2017-06-13

**Authors:** Sumit Karn, Madhu Dixit Devkota, Shahadat Uddin, Anne Marie Thow

**Affiliations:** 1 Food and Agriculture Organization of the United Nations, Kathmandu, Nepal; 2 Department of Community Medicine and Public Health, Institute of Medicine, Kathmandu, Nepal; 3 Complex Systems Research Group, The University of Sydney, Sydney, Australia; 4 Menzies Centre for Health Policy, School of Public Health, The University of Sydney, Sydney, Australia

**Keywords:** Nutrition, Policy, Actor, Nepal, IYCF

## Abstract

**Background:**

Despite concerted effort from government and partners, Nepal continues to have a high burden of under nutrition among children. Identifying opportunities to strengthen policy support for infant and young child feeding (IYCF) is a key component to improve child survival, growth and development. This study aims to explore policy support for IYCF and to identify the influential stakeholders for IYCF for effective future policy development and programmatic action.

**Methods:**

Policies relevant to IYCF were identified through web searches and direct approaches to relevant government ministries. Policy content was analysed based on four key domains focussed on mothers, using a qualitative synthesis approach. Three group interviews were conducted using the participatory tool “Net-Map”, to identify the influential stakeholders in IYCF policy and programming processes.

**Results:**

Twenty-six relevant policy documents were analysed for content relating to IYCF. General support for IYCF was found in most of the development plans and high-level health sector policies. Most implementation level documents included support for provision of correct information to mothers. Capacity building of frontline workers for IYCN and system strengthening were well supported through sectoral plans and policies. However, gaps were identified regarding maternity protection, support for monitoring and evaluation, and translation of high-level policy directives into implementation level guidelines, resulting in a lack of clarity over roles and responsibilities.

Both government and non-governmental stakeholders, particularly donors, emerged as influential drivers of IYCF policy decisions in Nepal, through technical assistance and funding. The Nutrition Technical Committee under the Ministry of Health, UNICEF, Suaahara, USAID and WHO were identified as key actors providing technical assistance. Key funding agencies were identified as UNICEF and USAID.

**Conclusions:**

This study reveals strong policy support for key dimensions of IYCF, supported by a highly networked stakeholder environment. Opportunities to further strengthen IYCF policy in Nepal include: further support for training of frontline workers and complementary feeding interventions; extending maternity leave provisions; and clarifying roles and responsibilities of actors, particularly non-governmental actors. Engaging technical and funding agencies and developing partnerships with other relevant actors will be crucial for ensuring effective policy translates into effective practice.

## Background

Nepal has achieved a significant reduction in mortality and morbidity amongst children under-five and women in the last decade [1]. Policy commitment to decentralisation and implementation of priority programs has improved access to maternal and child health services and contributed to achievement of most of the Millennium Development Goals (MDGs) [2]. Nepal is signatory to Agenda 2030, also known as the Sustainable Development Goals and has already adopted the 2012 WHA nutrition target in its recently endorsed health sector strategy [3].

Despite long-standing and influential cultural practices that support feeding babies with their mothers’ milk [4], poor feeding and care practices continue to be responsible for the high burden of under-nutrition amongst infants and young children in Nepal [5]. Recent survey data indicate low prevalence of early initiation of breastfeeding (44.5%), exclusive breastfeeding (69.6%), and age-appropriate complementary feeding practices [6]. Although the majority (66%) of infants between the ages of 6–8 months are introduced to complementary foods, the rate of minimum acceptable diet is very low. Only a quarter of children are fed the recommended breast milk or milk product, four or more food groups, and a minimum meal frequency according to their age and breastfeeding status [6]. While most of the data related to IYCF practices are far from reaching the 2012 WHA nutrition target, Nepal has already met the target for exclusive breastfeeding of up to or at least 50%. However, the recent decline in breastfeeding rate is a concern [7].

Globally, almost half of the deaths among under-5 children can be attributed to under-nutrition [8]. Global evidence shows a 45% reduction in risk of neonatal mortality if the baby is breastfed within 24 h of birth followed by exclusive breastfeeding [9]. Consumption of minimum acceptable diet (minimum meal frequency and adequate dietary diversity) is known to reduce the risk of under-nutrition in children [10]. Improving infant and young child feeding practices, therefore, has the potential to reduce mortality and morbidity, especially during the initial 2 years of life [11]. The World Health Organization (WHO) has recognised the protection, promotion and support of breastfeeding and optimal complementary feeding as crucial to improving child survival, growth and development [12].

The Government of Nepal has policies and programs in place to promote optimal practices for breastfeeding and complementary feeding, with the aim of achieving targets set by the WHO [13]. Nepal has adopted a multi-sector approach to addressing under-nutrition and has made a concerted effort to achieve policy coherence by involving all relevant sectors and concerned stakeholders. Recently, Ministry of Health and Population has also formulated a multisectoral policy advisory committee for nutrition [14], to provide technical as well as policy guidance for the health sector and beyond.

However, the 2012 World Breastfeeding Trend Initiative Nepal Report [15] identified gaps in IYCF policy and programs, scoring countries based on international recommendations for IYCF policy support. Nepal scored only 40.5 out of 100, compared to an average performance of 54.3 among the 40 countries assessed [16]. The report also observed that there has been little improvement in the IYCF policy environment since 2005.

Policy analysis enables us to understand and strengthen the policy environment [17, 18]. At this time, when a multi-sectoral approach is being given significant priority to address the multi-factorial dimensions of nutrition [9, 19], the role of stakeholders becomes particularly crucial in terms of understanding how the decision-making process is influenced for policy and programming.

This paper describes our analysis of the content of the policy documents for IYCF support and the stakeholders who influence IYCF policy and programme decisions in Nepal. Our study aimed to identify opportunities to strengthen the IYCF policy environment in Nepal and to identify the stakeholders best placed to achieve this. The results of this study will inform more effective policy development and programmatic action. First, the policy support for protection, promotion and support for breastfeeding and complementary feeding practices is described, followed by a participatory approach to analyse stakeholder’s influence on policy and program decisions.

## Methods

### Policy content analysis

The research team, through a series of consultative meetings of the South Asia Infant Feeding Research Network (SAIFRN), developed a standard matrix with the key IYCF domains that could be supported by policy, based on best-practice recommendations for promoting protecting and supporting IYCF. These included: a) general support for infant and young child nutrition, b) provision of correct information to mothers, c) training of health workers to counsel mothers, and d) enabling mother to engage with health care workers for informed decision making.

We used the definition by Buse et al. of “health policy” as embracing ‘courses of action (and inaction) that affect the set of institutions, organizations, services and funding arrangements of the health system but focussed on public (government) policy [20]. The SAIFRN research team used mind-mapping to identify four types of policy documents (and examples) with potential relevance to IYCF: those focussed on guiding overall government strategy; sector-specific documents; implementation-level documents; and other relevant policy documents.

We identified policy documents by searching relevant websites (National Planning Commission, Law Commission, Ministry of Health and Population, Department of Health Services, Ministry of Agricultural Development, UNICEF Nepal, World Bank-Nepal, USAID-Nepal). We used keywords such as “infant and young child feeding”, “nutrition”, “breastfeeding”, “complementary feeding”, “IYCF” “counselling” in various combinations. Reference lists found in the above documents were reviewed to identify potentially relevant reports and documents. We also contacted experts from government, external development partners, national and international non-governmental organisations, and academic institutions working in the field of health and nutrition to identify other relevant documents. Archives of organisations and newspapers were examined to identify information related to policy support for nutrition and IYCF.

The content of each policy was reviewed manually, using the matrix to identify provisions that support IYCF across each of the four domains described above. Relevant policy text and references were extracted and entered into the matrix template in an excel spread-sheet. The Extracts were grouped under the emerging themes. We used narrative as well as thematic synthesis to analyse the policy content in relation to IYCF support. We used a qualitative narrative synthesis approach to describe the ways in which the current policy landscape provided support for IYCF.

### Policy-relevant stakeholder analysis

In order to understand the role of actors, a participatory method known as Net-Map [21] was used. This interview technique investigates social networks, power relations and perspectives of relevant stakeholders. The tool can be used to visualise interactions between actors and how the network of these interactions influences decision-making.

The research team initially prepared an exhaustive list of stakeholders with knowledge and experience of IYCF policy processes in Nepal. Development of this list was informed by our knowledge of the field and discussions with expert informants including, nutrition programme managers at the Ministry of Health and Population, academic researchers, and colleagues working with the non-government organisations in the field of nutrition. The initial list comprised of people who had worked in this field previously, along with the current stakeholders. The list was subsequently revised and finalised by asking participants to identify further relevant participants. Those stakeholders not involved in recent policy development processes were left out.

We conducted 3 semi-structured group interviews between March and June 2015 involving a total of 25 participants (9 government, 5 academia/research, 5 UN agencies and 2 international non-government organisations, and 4 civil society organisations) to capture diverse views, opinions and perspectives (Table 1). The interviews focussed on the following questions: a) Who plays a role in shaping policy and program decisions on IYCF at the national level in the country?; b) Who provides funding and technical assistance to whom as a means of engaging in or influencing policy and programme decisions on IYCF?; and c) How strongly does each actor influence the shaping of policy and program decisions on IYCF? A trained facilitator led the interviews. Interviews were recorded and notes were taken by two to document inaudible features of the discussion. During these interviews, physical maps were created to represent participants’ descriptions of the stakeholders and their interactions, and qualitative data were recorded from the discussions.

**Table 1:** Participants who were interviewed using the NetMap method

NetMap Exercise	Total number of participants	Type of Participants
I *(March 24, 2015)*	9	Government: 3 (Department of Health Services, Child Health Division) Research/Academia: 2 (Padma Kanya Campus, New ERA, MIRA) Bilateral/Multilateral agencies: 3 (UNICEF, WFP, Micronutrient International) INGO: 0 Civil society organisation: 1 (Public Health Foundation)
II *(April 25, 2015)*	10	Government: 3 (Nutrition Section, CHD; Department of Health Services, Ministry of Health) Research/Academia: 3 (Kathmandu Medical College) Bilateral/Multilateral agencies: 2 (WHO, UNICEF) INGO: 1 (Save the Children) Civil society organisation: 2 (MaxPro, CSANN)
III *(June 13, 2015)*	6	Government: 3 (Child Health Division, Department of Food Technology and Quality Control, National Planning Commission) Research/Academia: 0 Bilateral/Multilateral agencies: 1 (UNICEF) INGO: 1 (Helen Keller International) Civil society organisation: 1 (Society for Public Health Physician in Nepal)

Ethics approval was obtained from Nepal Health Research Council for this study. Verbal consent was sought from participants during Net-Map interview after explaining the objective of the study and the exercise.

The quantitative Net-Map data from all three exercises were entered into MS Excel datasheet individually and later combined for analysis using social network analysis software ORA (Organizational Risk Analyzer, copyright Carley, Carnegie Mellon University) [22]. ORA was used to create a map visualising the key stakeholders and their networks for technical as well as the financial exchange for IYCF policy and programme decisions in Nepal. The qualitative data regarding opinions and perceptions of wide-ranging actors involved in IYCF were used to understand how these actors play a role influencing the decision for policy and programming for IYCF in Nepal.

In a Network Map, each stakeholder is represented by a node. Relationships between nodes is represented by a line, called a directed edge. Edges are described as directed because they indicate which node initiated the relationship (it’s direction). Directed edges also indicate the flow of support (in this case, funding and technical support). In our findings, we use four network centrality measures to describe the roles of actors in the network. *In-degree* indicates the number of directed edges incident on an actor, reflecting inward-directed support. *Out-degree* is the number of directed edges that originate at a node. *Betweenness* measures interconnectedness, and represents the capacity of an actor to control the flow of information between any pair of all other member actors in a network. *Closeness* measures the ease of reaching other nodes. An actor having high closeness centrality is well connected with the remaining network actors and vice versa.

## Results and discussion

### Policy content analysis

We identified a total of 26 relevant public policy documents. Most of these documents were from the health and agriculture sectors, while few policies belonged to the country’s strategic development plan. We also identified two acts of parliament. Content analysis indicated that policy support exists in Nepal for all four aspects of IYCF intervention examined (Table 2).

**Table 2:** Summary of policies supporting best practice intervention

Policy categories	# policy documents	General support for IYCF	Provision of correct information to mothers	Training of health workers to counsel mothers	Support mothers / caregivers to engage	Summary notes on how IYCF is supported in policy document
*High level support*						
Five-Year Development Plans (covering the period from 1965 to 2007 [23, 24, 52–55]	*6*	✓				• These strategic development plans of the country supports for better nutrition since the Fifth Strategic Development Plan. These plans focussed to provide nutrition education (counselling) through the peripheral health facilities on breastfeeding, raising public awareness and utilisation of nutritious foods to improve the nutritional status.
Three Year Interim Development Plan [56]	*1*	✓				• The recent interim development plan has given emphasis to focus to the pregnant and lactating women and mothers with children under 2 years of age through multi-sector nutrition plan, which covers comprehensive interventions on Infant and Young Child Feeding.
Multi-Sector Nutrition Plan 2013–2017 [27]	*1*	✓	✓	✓	✓	• This is the guiding plan for each sector to implement nutrition interventions and the major component of the plan is on implementation and scaling up of comprehensive maternal, infant and young child feeding program through BCC.
*Sector specific support*						
National Health Policy 1991, 2014 [30, 34]	*2*	✓				• The Health Policy of 1991 focused in the promotion of breast-feeding, growth monitoring, prevention of iodine deficiency disorders, iron and vitamin A deficiency, and health education to enable mothers to meet the daily requirements of children through locally available resources. The recently endorsed national health policy has given emphasis to food based approach (promotion of dietary diversity in complementary feeding) along with promotion for breastfeeding. Growth monitoring has been identified as a key delivery point for nutrition education.
Second Long Term Health Plan 1997–2017 [31]	1		✓	✓	✓	• Implementation of programmes to control micronutrient deficiencies (anaemia, vitamin A and iodine deficiency) in order to improve the nutritional status of women and children was emphasized. Nutrition supplementation, Nutrition education and rehabilitation were identified as major interventions under the essential health care services but there was no mention about infant and young child feeding.
Nepal Health Sector Program 2004–09, 2010–15 [32, 37]	*2*	✓	✓		✓	• These health sector reform strategies strongly put nutrition as one of the priority programmes in which nutrition counselling was given an emphasis to be provided during growth monitoring both at the health facility and community level. A strong priority is given to the promotion of improved feeding and health practices via the network of community-based health volunteers and health workers, as well as using media-based campaigns.
National Nutrition Policy and Strategy 2004 [35]	*1*	✓	✓	✓	✓	• This is the first guiding document for health sector in nutrition and protection, promotion and support for the optimal feeding practice for infants and young children was identified as one of the strategic objective of the document. Almost all aspects of IYCF has been included in the document.
National Health Communication Policy 2012 [33]	1					• Nothing has been mentioned on nutrition and IYCF in the policy.
National Safe Motherhood Policy 1998 [38]	*1*	✓	✓		✓	• Maternity care especially during ANC has emphasized counselling on breastfeeding and family planning. Promotion for early initiation of breastfeeding during delivery care is included.
National Policy on Skilled Birth Attendants 2006 [39]	*1*	✓		✓		• Assist women and their new-borns in initiating and establishing early and exclusive breastfeeding, including educating women and their families and other helpers in maintaining successful breastfeeding has been included as one of the core skills and abilities of Skilled Birth Attendants in the policy document.
Food and Nutrition Security Plan of Action 2013 [48]	*1*	✓	✓			• This plan document is a part of Agriculture Development Strategy which has broader support for improving the nutrition security and it has prioritized nutrition education and counselling on complementary feeding practices through promotion of dietary diversity.
*Implementation level support*						
National IYCF Strategy 2014 [13]	*1*	✓	✓	✓	✓	• The term “counseling’ has been emphasised in the document, however, lacks in actionable term. Strongly recommends to establish and roll out a national integrated community and harmonized counselling package for behaviour change on IYCF, hygiene and sanitation practices, stimulation and responsive feeding, care of the sick child and maternal nutrition and to strengthen IYCF counselling into key maternal and child health contact points. Gives priority to scale up of community based IYCF integrated with key health and nutrition community based programs and has clearly outlined for identification of effective approaches for harmonising IYCF components across health sector and beyond.
National Communication Strategy for MNCH 2012–16 [29]	*1*	✓	✓	✓		• Recognises the importance to protection, promotion and support for optimal feeding practice for infants and young children for action against protein energy malnutrition by building capacity on infant and young child feeding (IYCF) counselling. Behaviour change communication for changing dietary practices and raising awareness, on appropriate feeding practices has also been emphasised. The document has explicit objectives both for caretakers as well as service providers to influence the appropriate IYCF care and practices
National MIYCN Communication Plan 2016 [36]	*1*	✓	✓	✓	✓	• Use of all possible channels for raising awareness and emphasised the importance of interpersonal counselling at health facilities and outreach clinics.
*Other relevant policies*						
Breast Milk Substitute Act 1992 [28]	*1*	✓	✓		✓	• Has instructed to form a high-level Breast Feeding protection and promotion Committee under the chairmanship of Secretary of MoHP to supervise the compliance of this act and to protect and promote breast-feeding and regulate the sale and distribution of products. Provisions like following points are some of the highlights of the act- During the promotion and information about the complementary food/BM substitute, the benefits of BF and risks of substitutes must be included, the manufacturer or the distributor shouldn’t make contact with the general public in the premises of health care agency in order to enhance their business or such objectives, the label on any substitute products must include its method of use and must not discourage BF.
Nepal Labour Act 1992 [46]	*1*	✓				• A pregnant woman worker or employee shall be granted maternity leave with full pay for a total of 52 days before or after delivery. Such leave may be obtained not more than two times during the entire period of service, provided that in the event two children of a woman who has already utilized maternity leave twice do not survive and in the event that she becomes pregnant again, she may obtain maternity leave under this section upon the birth of two more children

### Broad guidance or support for IYCF

There is a strong commitment and priority given to nutrition, including infant and young child feeding, by the Government of Nepal. The 9th and 10th Five Year Plans, and the 1st Three-Year Interim Plan [23–25] specifically identify the importance of promoting breastfeeding, and the 2nd Three-Year Interim Plan [26] goes even further, including a recommendation to implement a programme on IYCF with a major focus on counselling.

This strategic support is reflected in the recent Multi-Sector Nutrition Plan [27], which brings together all relevant sectors. These include health, education, local development, agriculture, water, sanitation and hygiene and women and child welfare. Another key multi-sectoral policy, providing general support for good IYCF practices, is the Substitute for Breast Milk (Sale, Distribution and Control) Act of Nepal [28]. Its provisions include the formation of a high level Breast Feeding Protection and Promotion Committee under the chairmanship of the Secretary for Health. The committee includes representatives from health, agriculture, commerce and supply, industry, and education, demonstrating commitment to cross-sectoral policy coherence to protect, promote and support breastfeeding practices in Nepal. This committee is tasked not only to supervise compliance with the act but also more broadly to protect and promote breastfeeding.

### Provision of correct information to the mothers

Of the reviewed policy documents, 12 health sector specific policy and programmatic documents [13, 29–37] mandate the provision of correct IYCF information to mothers. These policies focus on ensuring the provision of information to mothers through counselling given by frontline health workers and health volunteers, and through dissemination of information, education and communication material, including via mass media.

The National Health Sector Program (NHSP I & II) includes specific strategies for promotion of counselling at the health facilities as well as through community level frontline workers, to increase awareness of recommended breastfeeding and young child feeding behaviours. The provision of counselling on breastfeeding, delivery care, nutrition and hygiene during antenatal clinics has been included in the Nepal Safe Motherhood Policy 1998 [38]. Likewise, the National Policy on Skilled Birth Attendants 2006 [39] also prioritises educating women and their families in the practice and support of successful breastfeeding practices. This includes encouraging family members to assist mothers to initiate and establish breastfeeding immediately after birth and practice exclusive breastfeeding; and supporting mothers and families to achieve this through inter-personal counselling provided by the birth attendants.

Provision of correct information to mothers through preparation and dissemination of information, education and communication materials and reaching mothers through different channels of communication is recommended in the Multi-Sector Nutrition Plan 2012 [27]. Similarly, the NHSP-II [40] identifies improved infant feeding and health practices through community networks and media based campaigns as priorities. Likewise, the Substitute for Breast Milk (Sale, Distribution and Control) Act of Nepal [28] protects breastfeeding by restricting the promotion of breast-milk substitutes. However, neither of these documents addresses the promotion of age-appropriate complementary feeding. Moreover, the National Health Communication Policy 2012 [33] does not include any policy direction or strategies for IYCF communication. The national IYCF Strategy [13] prescribes a standard set of messages for dissemination through all possible channels across all sectors to ensure uniformity and consistency of the information provided to the target groups.

### Training and capacity building of workers on IYCF

Four policies [13, 27, 35, 41] provide for the capacity development of workers for nutrition, including IYCF. At a strategic level – and recognising the importance of a multi-sector approach – the recently endorsed national Multi-Sector Nutrition Plan (MSNP) [27] has outlined a comprehensive and systematic approach to building the capacity of human resources of all relevant sectors. These sectors include health, education, local development, agriculture, water, sanitation and hygiene, and women and child welfare in nutrition. Specifically, this plan focusses on providing counselling in support of adequate maternal, infant and young child nutrition. The strategy also includes capacity assessment [42] on nutrition for frontline workers, district level managers and central staff from all relevant sectors; developing a capacity building plan and carrying out training for nutritional and non-nutritional professionals from all the sectors focusing on local level cadres. However, the roles of each sector in conducting training needs assessment for nutrition are not specified in the document. It also specifies the different layers of training to the service providers. However, the training focuses on management skills rather than developing a skilled counselling workforce. Training to build capacity of health workers, community level service providers and medical professionals for counselling at the health facilities is mandated within the National Nutrition Policy and Strategy in 2004 [35]. The Ministry of Health delivers this training with support from professional bodies and academic institutions.

The strategic guidance of MSNP [27] on scaling up of community IYCF interventions is operationalized in the recently drafted IYCF Strategy [13], which includes development of training guidelines and modules for health facility as well as community level frontline health workers. The modules on breastfeeding are aligned with the global training manuals developed by WHO and UNICEF [43, 44]. The IYCF Strategy [13] calls for the development of standard training manuals with sessions that focus on the promotion of optimal breastfeeding practices (early initiation of breastfeeding, exclusive breastfeeding and extended breastfeeding), timely initiation of complementary feeding and, age-appropriate complementary feeding practices to support adequate quantity and quality of complementary feeding.

The national IYCF Strategy [13] also explicitly identifies the need to provide practical skills and knowledge to frontline health workers to enable them to identify and manage breastfeeding problems and empower mothers to overcome barriers to adopting optimal feeding and care practices. In Nepal, frontline health workers include those working in the health facilities at the community level as well as female community health volunteers. The strategy emphasises the importance of skill-based training to enhance counselling skills of community level service providers from all relevant sectors in line with the MSNP [27]. The document fails to identify the community level cadres from non-health sectors to be trained to provide counselling on nutrition. Using these non-health cadres as peer counsellors, social mobilisers and agriculture extension workers would also strengthen the capacity of sectors other than health to strengthen nutrition and institutionalise nutrition in non-health systems. The strategy includes a recommendation for the review of existing training materials in order to harmonise and integrate into a single comprehensive set of IYCF and Baby Friendly Hospital Initiative (BFHI) training materials for pre-service as well as in-service training for health and non-health service providers. To implement the maternal as well as IYCF Strategy, a joint plan of action for maternal, infant and young child nutrition [41] has recently been drafted.

While both the MSNP [27] and the national IYCF Strategy [13] have recommended a comprehensive and standardised IYCF training package, our review did not identify a clear statement on the roles and responsibilities of relevant agencies or the implementation modalities for the training of service providers. Detailed training schedules, content, resources and personnel, well defined roles and responsibilities and the criteria for selection of participants could be integrated into the national IYCF strategy [13] and the National Plan of Action for MIYCN [41], which are yet to be endorsed by the Ministry of Health and Population.

### Enabling mothers to engage with care

Eight of the health sector policy documents and the recent Multi-Sector Nutrition Plan [13, 27, 28, 31, 32, 35–38] target mothers and care takers for IYCF support. Mothers (and caregivers) are specifically enabled to engage with care related to IYCF through improving access to health services for marginalised populations, engaging women in local governance, enabling employed women access to maternity leave and flexible working arrangements, and establishing community based support services to augment formal health service provision.

Empowering and enabling mothers and care takers by involving them in training and group meetings for counselling, through social mobilisation for behaviour change, and improving access to IYCF services is the major approach outlined in the MSNP [27]. Given increasing disparity in IYCF practices, the plan mandates socially inclusive, gender and child friendly approaches in the design and implementation of its programmes; it focuses on reaching the most marginalized, disadvantaged and poorest segments of the population. For women not working in formal sectors, the IYCF strategy provides community-based support through the establishment of mother-to-mother support groups, involving male partners, peer educators or other family and community members. The program is currently being scaled up in 41 out of 75 districts.

The National Nutrition Policy and Strategy in 2004 [35] has identified as one of its strategies, the provision to promote mother and child friendly working environments, by establishing crèches, promotion of breastfeeding breaks for working mothers and to advocate for extended maternity as well as paternity leave with policy makers and central level managers. The recently drafted IYCF Strategy and its Plan of Action [13, 41] have identified the importance of the provision of breastfeeding break and workplace day care centre. These policies align with recommendations by the WHO/UNICEF [43, 45]. However, the detailed activities for its implementation have not been specified.

The current provision for maternity leave, in the Labour Act [46], which is important for establishing and continuing breastfeeding, differs for women working in different sectors. The duration of leave entitlement varies from 98 days of paid maternity leave for a woman judge at the District Court or Appellate Court, to only 45 days for a woman working at a tea plantation. The Government of Nepal has also decided to grant paternity leave [47]. Under this provision, the male employees could be given 11 days paid leave for the birth of each of their first two children. The recent IYCF strategy [13] document has explicitly mentioned the need for a review and amendment of the national legislation on maternity protection. It has also suggested implementing a model intervention to demonstrate effective implementation of the revised law in selected private and public sectors and thereby scaling it up nationwide by 2020 based on the effectiveness. However, it is important to note that these provisions apply only to women working in the formal sector, and that the current provision of maternity leave for 12 to 14 weeks is not applied uniformly in the private sectors [47]. More importantly, the majority of women work in the informal sector and are excluded from the maternity leave benefits.

### IYCF support across other sectors

We also identified support for IYCF at the policy level in Nepal in the form of strong multisectoral collaboration, and support for agriculture and food security. The Multi-Sector Nutrition Plan [27], Food and Nutrition Security Plan of Action [48], National IYCF Strategy [13] and Health Sector Strategy for Addressing Maternal Under-nutrition [49], seek to integrate IYCF related services with the programme of other sectors. For example, the Multi-Sector Nutrition Plan [27] emphasised the integration or linking of IYCF with other sectoral programmes such as the Child Cash Grant (administered by Ministry of Local Development), and water, sanitation and hygiene programmes (administered by Ministry of Urban Development), as well as ensuring that support for good IYCF is explicit in other health sector programmes such as antenatal care, birth preparedness package and growth monitoring.

While there has been limited focus on complementary feeding in the health sector policies, dietary diversity is supported by the Food and Nutrition Security Plan of Action [48] under the Agriculture Development Strategy [50]. This strategy provides specific programmes to increase access to safe, nutritious and diversified food, and to address food and nutrition security in the existing agriculture education system for production diversification and nutrition diversity. The National Nutrition Policy, MSNP and National IYCF Strategy [13, 27, 35] have provided a detailed framework for monitoring and evaluation of programmes, including [13] direction to include it in regular surveillance of health and nutrition in the country.

### Stakeholders and social network analysis

#### Overview of findings

**Fig. 1:**
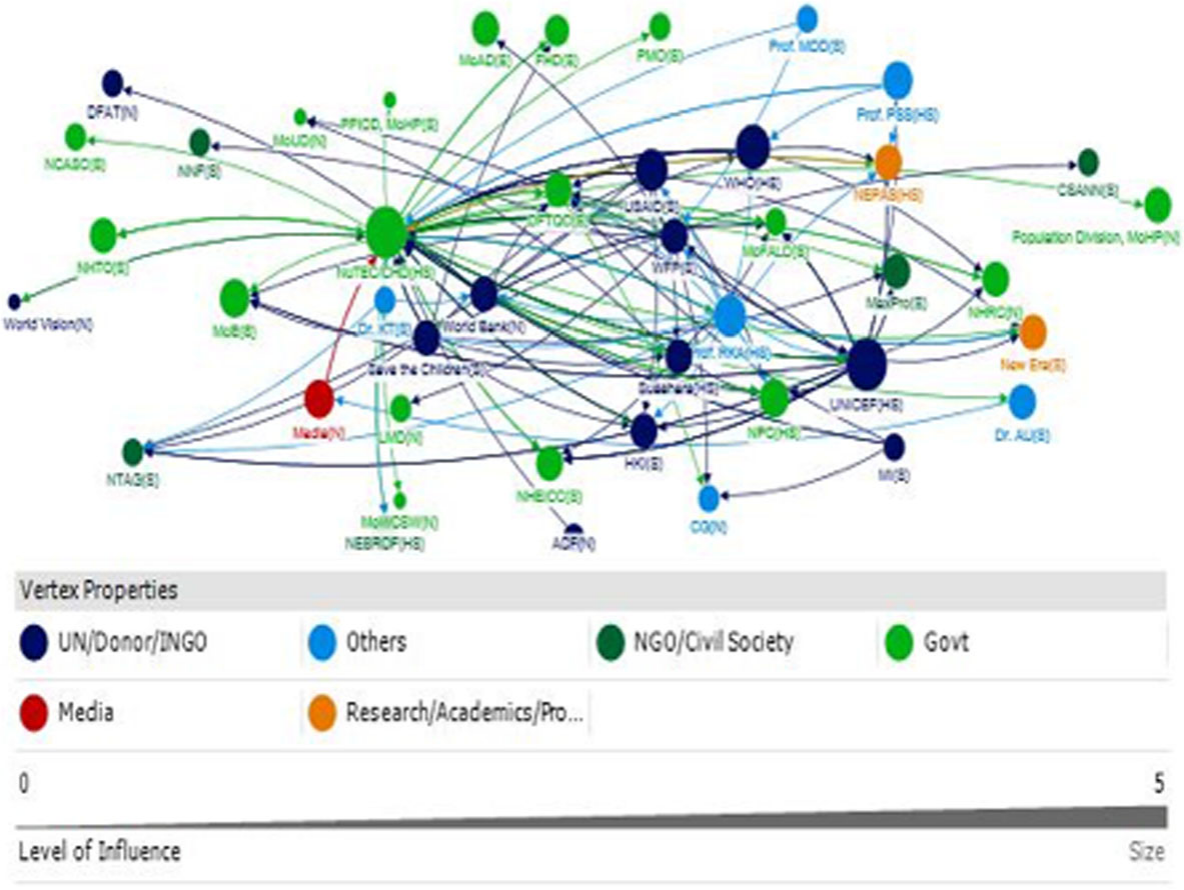
Social network of stakeholders for provision of technical assistance for IYCF policy and programming in Nepal

**Fig. 2:**
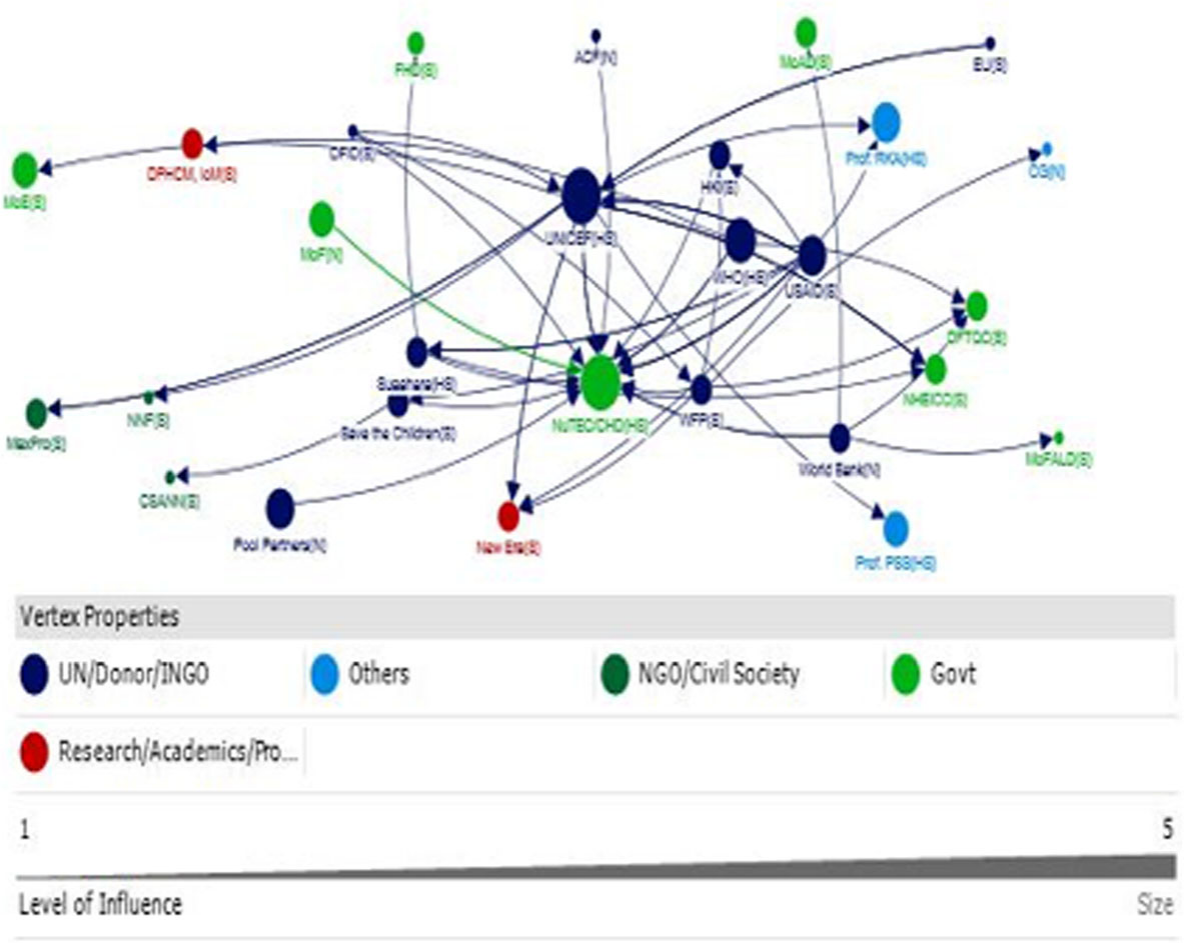
Social network of stakeholders for provision of funding for IYCF policy and programming in Nepal

A total of 43 actors were identified through three Net-Map group interviews. Six categories of stakeholders were identified, including: Government; Research, Academic and Professional Bodies; UN, Donor, INGO; NGOs and Civil Society Organisations; and Others. These stakeholders were connected through flows of funding and technical advice, represented by lines (Figs. 1 and 2). The varied size of the nodes represents the relative level of influence of the actors in terms of policy and programmatic decision for IYCF at the country level ranging from a smallest node as least influential to the largest node as the most influential.

#### Tracing the central stakeholders

Several actors were found to be highly influential through the provision of technical assistance (Fig. 1; Table 2). The Nutrition Technical Committee (NuTEC) at the Nutrition Section, Child Health Division (CHD) was the primary influencer in the network. This is a committee under the MoHP to provide policy advisory and technical as well as programmatic guidance to health sector for nutrition. The quantitative analysis indicates that NuTEC received substantial technical support (in-degree), provided technical support (out-degree), was accessible to a wide range of other actors in the network (closeness), and exerted a high degree of control over the flow of information between actors by its position in the network (betweenness) (Table 3). This was supported by the (qualitative) discussion regarding NuTECs central position as an actor. The NuTEC plays a key role in providing technical input as well as receives the input for IYCF policy and programmatic decision. Suhaara, an NGO, was the only other actor to rank in the top 10 for all of the four network measures, also indicating its role as an influencer in the network.

**Table 3:** List of identified stakeholders with their centrality and network measures (in-degree, out-degree, closeness and betweenness centrality)

Funding				Technical support			
*In-degree*	*Out-degree*	*Betweenness*	*Closeness*	*In-degree*	*Out-degree*	*Betweenness*	*Closeness*
NuTEC/CHD (0.4286)	UNICEF (0.2857)	NuTEC/CHD (0.5533)	NuTEC/CHD (0.0238)	NuTEC/CHD (0.4419)	NuTEC/CHD (0.5581)	NuTEC/CHD (0.6370)	NuTEC/CHD (0.0200)
DFTQC (0.1071)	USAID (0.2500)	UNICEF (0.3775)	UNICEF (0.0200)	DFTQC (0.1395)	UNICEF (0.3488)	UNICEF (0.1226)	UNICEF (0.0140)
New Era (0.1071)	WFP (0.1786)	World Bank (0.1469)	USAID (0.0192)	NPC (0.1395)	Prof. RKA (0.3256)	WFP (0.1133)	Prof. RKA (0.0140)
UNICEF (0.1071)	World Bank (0.1429)	WFP (0.1119)	DFID (0.0179)	HKI (0.1163)	Save the Children (0.1860)	Save the Children (0.0590)	WFP (0.0130)
HKI (0.0714)	Suaahara (0.1071)	USAID (0.0981)	WFP (0.0167)	NEPAS (0.1163)	WFP (0.1628)	Prof. RKA (0.0524)	Save the Children (0.0130)
NHEICC (0.0714)	DFID (0.1071)	Suaahara (0.0883)	Suaahara (0.0164)	NTAG (0.1163)	USAID (0.1628)	World Bank (0.0510)	DFTQC (0.0130)
Prof. RKA (0.0714)	WHO (0.1071)	WHO (0.0807)	World Bank (0.0161)	MoE (0.0930)	World Bank (0.1395)	DFTQC (0.0216)	World Bank (0.0120)
CG (0.0357)	Save the Children (0.0714)	Save the Children (0.0688)	HKI (0.0161)	MoFALD (0.0930)	Suaahara (0.1163)	Suaahara (0.0216)	Suaahara (0.0120)
CSANN (0.0357)	HKI (0.0357)	DFTQC (0.0245)	Save the Children (0.0159)	New Era (0.0930)	WHO (0.0930)	MI (0.0078)	USAID (0.0120)
DPHCM, IoM (0.0357)	ACF (0.0357)	New Era (0.0200)	WHO (0.0156)	Suaahara (0.0930)	Dr. KT (0.0930)	USAID (0.0069)	WHO (0.0120)

*Abbreviations: NuTEC/CHD* Nutrition Technical Committee/Child Health Division, *UNICEF* United Nations Children Fund, *Prof RKA* Prof. Ramesh K Adhikari, *WFP* World Food Programme, *DFTQC* Department of Food Technology and Quality Control, *USAID* United States of America for International Development, *WHO* World Health Organization, *Dr. KT* Dr. Kalpana Tiwari, *NPC* National Planning Commission, *MoE* Ministry of Education, *MoFALD* Ministry of Federal Affairs and Local Development, *HKI* Helen Keller International, *NEPAS* Nepal Paediatrics Society, *Prof. PSS* Prof Prakash Sunder Shrestha, *NHRC* Nepal Health Research Council, *NHEICC* National Health Education, Information and Communication Centre, *MI* Micronutrient International, *NTAG* Nepal Technical Assistance Group, *Prof. MDD* Prof Madhu Dixit Devkota, *Dr. AU* Dr. Aruna Upreti, *NHTC* National Health Training Centre, *ACF* Action Against Hunger, *FHD* Family Health Division, *NEBROF* Nepal Breastfeeding Promotion Forum, *MoWCSW* Ministry of Women, Child and Social Welfare, *NCASC* National Centre for AIDS and STD Control, *PMO* Prime Minister Office, *MoHP* Ministry of Health and Population, *PPICD* Policy, Planning and International Cooperation Division, *CG* Chaudhary Group, *MoUD* Ministry of Urban Development, *NNF* Nepal Nutrition Forum, *CSANN* Civil Society Alliance for Nutrition in Nepal, *DFAT* Department of Foreign Aids and Trade, *LMD* Logistic Management Division, *MoAD* Ministry of Agricultural Development

International agencies – UNICEF, Save the Children, World Bank and USAID – were also key sources of technical support (out-degree), and were strategically positioned within the network (betweenness and closeness) (Fig. 1; Tables 2 and 3). In addition to these organisations, one individual (an academic) emerged as an important source of technical assistance for IYCF.

The most influential actors with respect to provision of funding were NGOs, mainly INGOs (Out-degree; Table 3), and the network map showed a funding flow from INGOs towards government departments (Fig. 2). The primary influencer with respect to funding was UNICEF, which found to provide funding (out-degree), coordinate funding from other donors (in-degree), was accessible to a wide range of other actors in the network (closeness) and exerted a high degree of control over the flow of funds between actors by its position in the network (betweenness) (Table 3). WHO, the World Bank, Suaahara, Save the Children, USAID and WFP emerged as secondary influences, providing funding (out-degree) and having high connectivity within the network (closeness and betweenness).

NuTEC, Suaahara and UNICEF were notable actors in that they were influential in both the funding and technical support networks – each of these actors appeared in the top ten for seven of the eight network measures (Table 3). The discussion indicated that this reflects their roles as coordinators of IYCF policy and program action. The main differences apparent between these three was that UNICEF was not one of the top ten receivers of technical support, whereas Suaahara was not one of the top ten receivers of funding, and NuTEC was not one of the top ten providers of funding.

In terms of relative influence for IYCF policy and programme decisions, the (qualitative) discussion indicated that government agencies namely NuTEC/CHD, NHTC, NHEICC, NPC, DFTQC, FHD were perceived as the most influential by the interview participants because they had the authority and the decision-making power. UNICEF, WHO, USAID, Save the Children and Suaahara were also influential in terms of providing technical support and funding. Individuals like Prof PSS, RKA and AU were perceived to be influential through their provision of technical support and high level of connectedness.

#### Communities of actors

Further analysis of the social network revealed that there were distinct communities within the broader network for technical support as well as financing. Actors within each of these of IYCF policy and program communities were all more engaged among themselves compared to other actors.

**Fig. 3:**
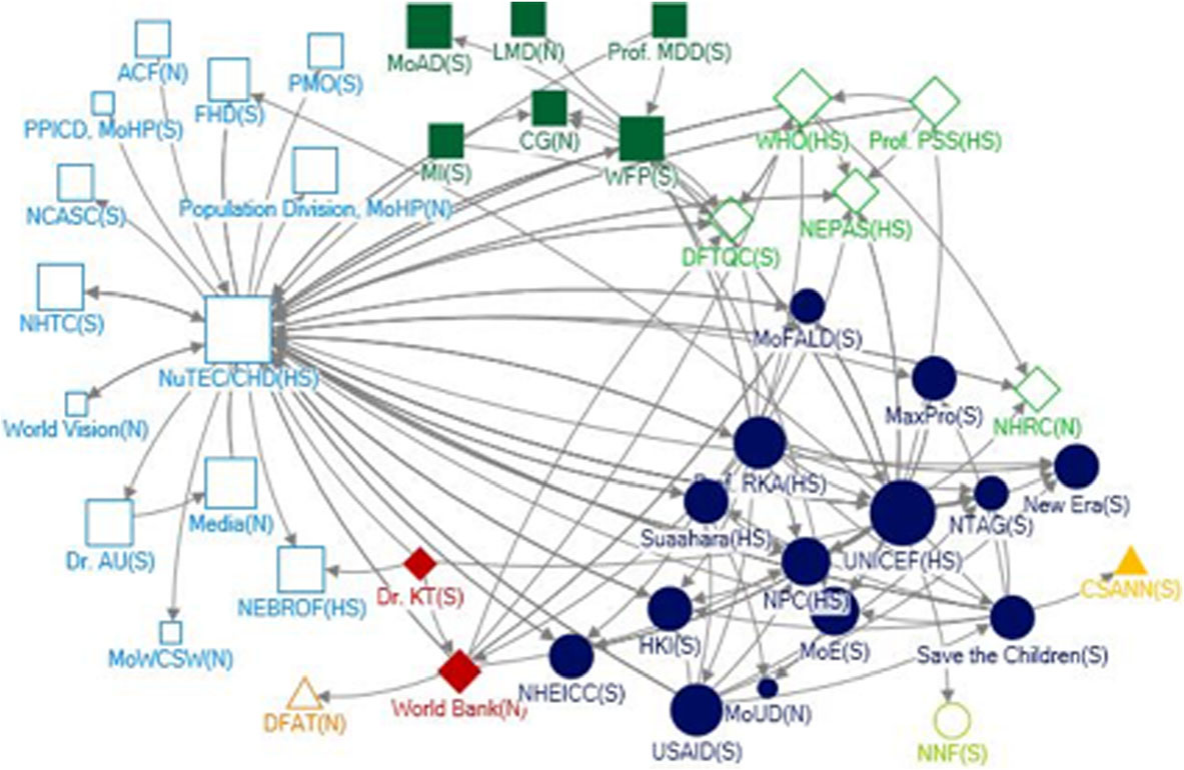
Technical assistance community under the social network of stakeholders for IYCF policy and programming in Nepal

There were four distinct major communities under the technical assistance network (Fig. 3). The composition of these technical communities was diverse; all included a mix of actors from government, INGOs, donor agencies and academic institutions (Fig. 1; Fig. 3). Government agencies dominated the largest community, which had NuTEC in the centre, and NuTEC was found to be connected with all other communities. Another major technical assistance community had mostly INGOs and donor agencies with Suaahara, UNICEF and Prof RKA as the key actors, and Suaahara and UNICEF were also found to have connection across to other communities. The WFP was at the centre of a third major community, and WHO and DFTQC were central players in a fourth, smaller, community. There were a few small communities with two or one actors that were not linked with all the communities in the network. This analysis of communities indicates that NuTEC plays an essential role as a hub for technical assistance from different communities. This understanding of working patterns and grouping of actors can also help to inform advocacy efforts.

**Fig. 4:**
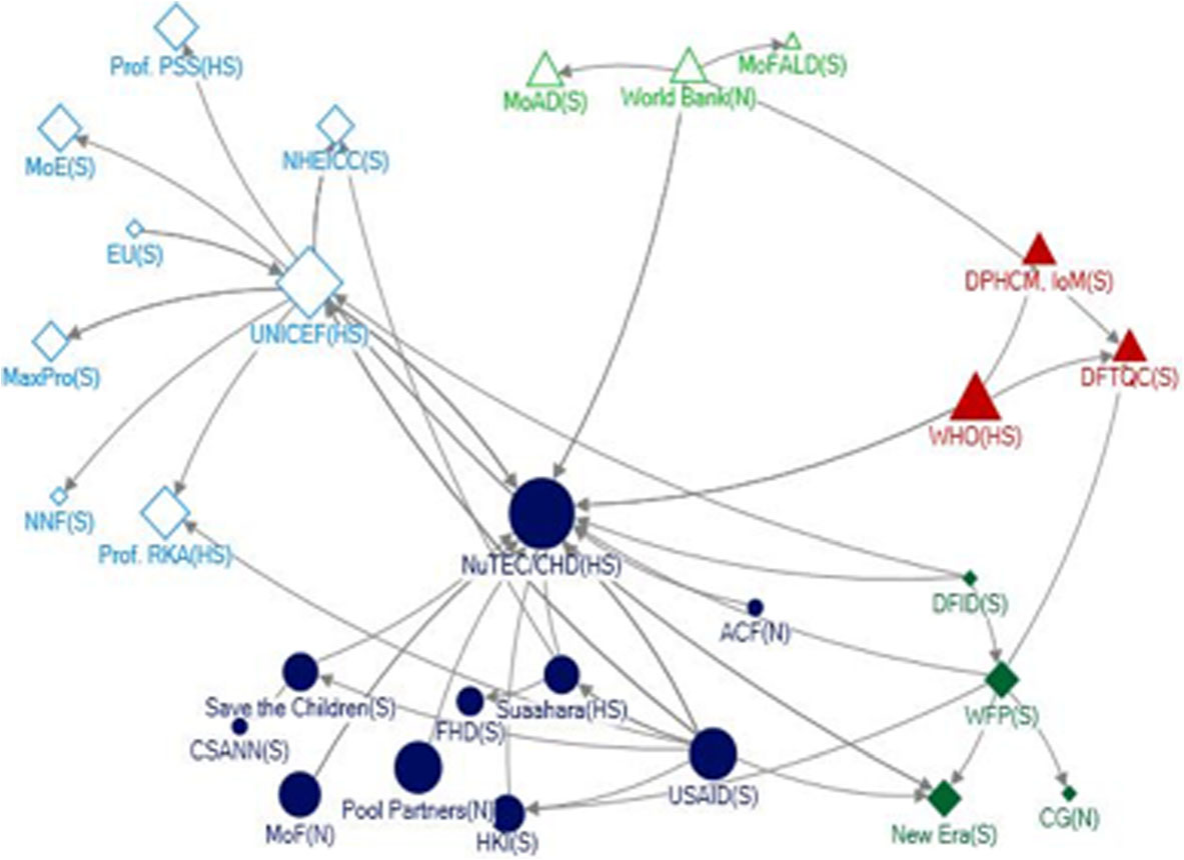
Funding community under the social network of stakeholders for IYCF policy and programming in Nepal

Five communities emerged within the funding network (Fig. 4). Each community was clustered around one or two major donors (USAID and Pool Partners; UNICEF; World Bank; WFP; and WHO). NuTEC received funding support from all donor agencies from these different communities, and were part of a community containing several other donors. In other communities, local NGOs, INGOs and research institutions were the major recipients of funding support. The World Bank and WHO provided funding support to government institutions only.

## Conclusion

The analysis indicates that the effort for strengthening the policy landscape for promotion, protection and support for optimal IYCF practices in last few years have led to a strong and supportive policy environment for IYCF in Nepal. Current initiatives by government to harmonise the interventions across sectors is a particularly positive step. This policy focus has been reflected in stakeholder influence. Multisectoral nutrition committees play a central role in drafting the IYCF strategy and National Plan of Action [13, 41] and review the evidence to inform reformulation of programming.

Specific strengths in the policy landscape that were identified in our analysis. These include support for counselling to mothers and care takers, creating enabling environment for breastfeeding, ensuring correct and consistent information for mothers through all possible contact points and health system support for IYCF, building the capacity of frontline workers for IYCF counselling and creating supportive environment for mothers to seek care and support for improving IYCF.

However, we observed a gap in the policy and programming environment around complementary feeding. Given the current low level of complementary feeding practices in Nepal [6], addressing this gap should be prioritised.

This research has identified specific opportunities to improve IYCF policy in Nepal, as well as identifying key stakeholders to inform advocacy, using policy mapping and stakeholder analysis methods. Consideration of two key aspects of policy (content and actors) through in-depth content analysis and stakeholder analysis is a strength of this study. The study has considered crucial domains of IYCF in the analysis, which will guide to address the gaps identified in terms of effective IYCF policy and interventions. However, the limitations of this research include the decision not to measure changes in the policy environment and stakeholder environment over time (due to funding constraints), and the focus on only two avenues of influence in the stakeholder mapping. In addition, the detailed study to understand the power dynamics and factors responsible for an actor to be influential in the network would provide a better understanding about the IYCF policy landscape in the country.

The recently drafted strategy on IYCF [13] makes provision for effective counselling at the health facility and community level, for which, it has emphasised the importance of capacity building of health workers and the role of health system support in institutionalising IYCF programmes. This IYCF programme has been rolled out in most of the districts [51]. However, there are significant opportunities to enhance the realisation of these policy goals through the inclusion of more specific attention to implementation. The National Plan of Action on MIYCN [41] seeks to prioritise IYCF counselling and supporting interventions through the health system, but lacks clarity around roles and responsibilities of partners, effective mechanisms for monitoring and evaluation at national level, and allocation of sufficient resources. Opportunities to strengthen IYCF policy include the development and consistent implementation of standard training packages for frontline workers, and improving monitoring and evaluation of IYCF policies and programs.

Given increasing urbanisation and more women coming into the formal workforce, another opportunity to strengthen policy is to enhance support for working mothers, particularly in urban areas. This analysis indicates that specific opportunities exist to extend maternity leave for 6 months for women in formal employment, and to strengthen the community-based efforts to provide access to health services for women in informal employment. Our stakeholder analysis suggests that effective advocacy will require collaboration between government and non-government stakeholders to develop and propose evidence based policy options. It would be important for the major actors, such as UNICEF and NuTEC, to strengthen links with the actors responsible for the different aspects of maternity protection, such as the Ministry of Labour and private sector actors, who are not well connected in the network so that their perspectives could also be considered during policy decisions and programming.

A major challenge identified for implementation of nutrition interventions, including IYCF, are the shortage of human resources and their capacity in delivering nutrition services at the community level [42]. The quality of counselling on IYCF largely depends on the knowledge and skills of frontline health workers. The Government of Nepal is responding to this challenge, with MoHP initiating the development of an integrated training package on MIYCN [13]. However, our content analysis identified a need for this initiative to be supported by clear timelines and delineation of roles and responsibilities. The stakeholder analysis component of this study further supports this, indicating that implementation of nutrition interventions, including IYCF, is heavily dependent on non-government sectors, yet their roles and responsibilities are not clearly defined in the policy documents. The MSNP [27] also does not highlight training the frontline workers from other sectors on IYCF.

One option may be to explore alternate community-level cadres (like peer counsellor, social mobilisers, agriculture extension workers) to deliver the IYCF counselling other than health frontline workers. Given there is increasing commitment to multi-sector approaches to nutrition in Nepal, building capacity of such cadres could improve delivery of nutrition services. This approach has been included in the policy [48] under the agriculture sector. This approach would build on the increasing role of NGOs in IYCF policy delivery.

## Abbreviations

IYCF: Infant and Young Child Feeding
IYCN: Infant and Young Child Nutrition
USAID: United States Agency for International Development
WHO: World Health Organisation
UNICEF: United Nations Children Fund
CHD: Child Health Division
MDG: Millennium Development Goal
SAIFRN: South Asia Infant Feeding Research Network
MIYCN: Maternal Infant and Young Child Nutrition
MNCH: Maternal, Newborn and Child Health
NHSP: Nepal Health Sector Programme
ANC: Ante Natal Care
MSNP: Multi-Sector Nutrition Plan
UN: United Nations
INGO: International Non-Governmental Organisation
NGO: Non-Governmental Organisation
NuTEC: Nutrition Technical Committee
WFP: World Food Programme
HKI: Helen Keller International
NHTC: National Health Training Centre
NPC: National Planning Commission
NHEICC: National Health Education, Information and Communication Centre
DFTQC: Department of Food Technology and Quality Control
FHD: Family Health Division
PSS: Prakash Sunder Shrestha
RKA: Ramesh Kant Adhikari
AU: Aruna Upreti
MoE: Ministry of Education
MoAD: Ministry of Agricultural Development
